# Cavitating mesenteric lymph node syndrome: a rare complication of celiac disease—case report and literature review

**DOI:** 10.1093/omcr/omag041

**Published:** 2026-04-14

**Authors:** Taha Yassine Aaboudech, Sabrine Derqaoui, Siham Mesmoudi, Manal Cherkaoui, Ahmed Jahid, Ikram Errabih, Zakia Bernoussi, Kaoutar Znati

**Affiliations:** Pathology Department, Ibn Sina Hospital, Lamfadel Cherkaoui street, Rabat 10100, Morocco; Mohammed V University in Rabat, Rabat, Morocco; Pathology Department, Ibn Sina Hospital, Lamfadel Cherkaoui street, Rabat 10100, Morocco; Mohammed V University in Rabat, Rabat, Morocco; Pathology Department, Ibn Sina Hospital, Lamfadel Cherkaoui street, Rabat 10100, Morocco; Mohammed V University in Rabat, Rabat, Morocco; Pathology Department, Ibn Sina Hospital, Lamfadel Cherkaoui street, Rabat 10100, Morocco; Gastroenterology Department, Ibn Sina Hospital, Rabat, Morocco; Pathology Department, Ibn Sina Hospital, Lamfadel Cherkaoui street, Rabat 10100, Morocco; Mohammed V University in Rabat, Rabat, Morocco; Pathology Department, Ibn Sina Hospital, Lamfadel Cherkaoui street, Rabat 10100, Morocco; Gastroenterology Department, Ibn Sina Hospital, Rabat, Morocco; Pathology Department, Ibn Sina Hospital, Lamfadel Cherkaoui street, Rabat 10100, Morocco; Mohammed V University in Rabat, Rabat, Morocco; Pathology Department, Ibn Sina Hospital, Lamfadel Cherkaoui street, Rabat 10100, Morocco; Mohammed V University in Rabat, Rabat, Morocco

**Keywords:** cavitating mesenteric lymph node syndrome, celiac disease, malabsorption, lymphadenopathy, gluten-free diet

## Abstract

Background: Cavitating Mesenteric Lymph Node Syndrome (CMLNS) is an uncommon complication of celiac disease, characterized by cystic mesenteric lymph nodes and high morbidity. Case Presentation: A 46-year-old male with chronic diarrhea, vomiting, and weight loss had hypoalbuminemia and elevated anti-TTG IgA. Duodenal biopsy confirmed celiac disease (Marsh IIIb). CT showed pseudocystic mesenteric lymph nodes and splenic atrophy, initially suggesting malignancy. Laparoscopic excision revealed cavitated lymph nodes with liquefactive necrosis and an inflammatory rim, excluding lymphoma or infection. The patient received a gluten-free diet, nutritional support, and corticosteroids, achieving clinical improvement and stable imaging at six months. Conclusion: CMLNS should be considered in celiac patients with refractory symptoms and atypical imaging. Early recognition through clinical, radiological, and pathological assessment, combined with a gluten-free diet and supportive care, is crucial to improve outcomes.

## Introduction

Cavitating Mesenteric Lymph Node Syndrome (CMLS) is a rare and underrecognized complication of celiac disease, characterized by cystic or cavitating changes in mesenteric lymph nodes.

We present the case of a 46-year-old man with a three-year history of chronic diarrhea, recurrent vomiting, abdominal pain, and significant weight loss. Despite extensive evaluations, no definitive diagnosis had been made. Imaging revealed multiple retroperitoneal lymphadenopathies. Histopathological analysis ultimately confirmed celiac disease with associated CMLS.

This case emphasizes the importance of integrating clinical, radiological, and histological findings when evaluating atypical presentations of celiac disease.

## Case report

We report the case of a 46-year-old male with no notable medical history, who presented with a three-year history of progressively worsening malabsorptive syndrome. The clinical presentation included persistent watery diarrhea, postprandial vomiting, anorexia, asthenia, and a 13 kg weight loss over two years. Intermittent polyarthralgia affecting both small and large joints was also noted. Physical examination revealed features of severe protein-energy malnutrition, including a BMI of 12.86 kg/m^2^, bilateral lower limb edema, and a mildly distended, non-tender abdomen.

Laboratory evaluation showed marked hypoalbuminemia (16.8 g/l), hypoproteinemia (48 g/l), hypocholesterolemia, and folate deficiency. C-reactive protein was elevated (49 mg/l), reflecting systemic inflammation. Serologic testing revealed significantly elevated anti-tissue transglutaminase (anti-TTG) IgA antibodies (96.37 IU/ml), supporting the diagnosis of celiac disease. Duodenoscopy revealed fissured and nodular mucosa, and histologic examination of duodenal biopsies demonstrated subtotal villous atrophy, crypt hyperplasia, and increased intraepithelial lymphocytes (Marsh–Oberhuber stage IIIb), consistent with active celiac enteropathy.

**Figure 1:**
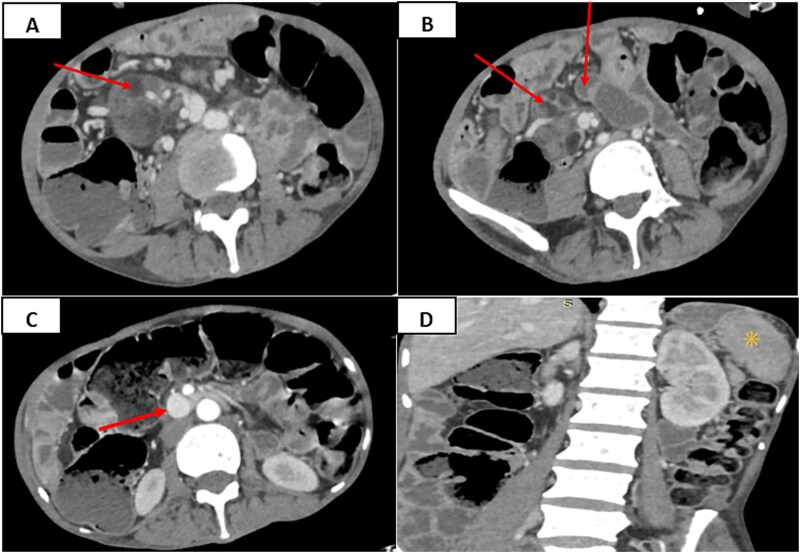
(A, B) axial contrast-enhanced abdominal CT images show conglomerate mesenteric lymph nodes with fat-fluid levels and pseudocystic centers (arrowheads). (C) Axial contrast-enhanced view demonstrates dilation of the superior mesenteric vein, reflecting venous congestion secondary to lymphadenopathic compression (arrowhead). (D) Coronal contrast-enhanced CT image reveals splenic atrophy (asterisk).

Abdominopelvic CT initially raised concern for a gastrointestinal stromal tumor due to multiple mesenteric masses. However, further thoracoabdominopelvic imaging revealed jejunalization of the distal ileum, splenic atrophy, and multiple mesenteric and ileocolic lymphadenopathies, some with hypodense, pseudocystic centers (Fig. 1). A diagnostic laparoscopy with excision of a mesenteric nodule was performed for further evaluation.

**Figure 2:**
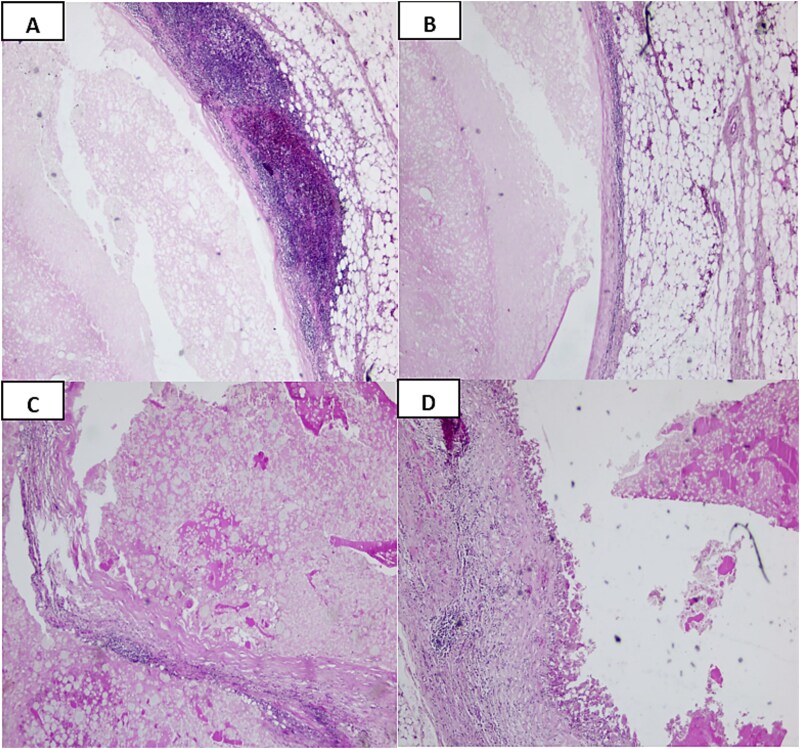
(A, B) H&E-stained slides of a lymph node with a central cavitation filled with pale eosinophilic, acellular material consistent with liquefactive necrosis (a: ×4; B: ×10 magnification). (C, D) H&E-stained slides demonstrating necrotic material surrounded by dense lymphohistiocytic inflammation with foamy histiocytes and a mantle of small lymphocytes, forming a characteristic rim (×20 magnification).

Gross pathological analysis of the resected tissue (3.5 × 2.0 × 1.2 cm) revealed two friable, pearly nodules with necrotic appearance. Microscopic examination showed three lymph nodes within mesenteric fat, two exhibiting central cavitation filled with pale eosinophilic acellular material consistent with liquefactive necrosis. These were surrounded by dense lymphohistiocytic inflammation with foamy histiocytes and a peripheral mantle of small lymphocytes, forming a characteristic inflammatory rim. No granulomas, atypia, or malignancy were identified. Special colorations and cultures excluded mycobacterial or fungal infections. Immunohistochemistry showed CD68-positive histiocytes, with negative S100 and langerin staining, excluding histiocytosis (Fig. 2).

These features confirmed the diagnosis of CMLNS, a rare complication of longstanding or refractory celiac disease. The underlying mechanism is thought to involve immune-mediated vascular injury resulting in lymph node ischemia and necrosis. No evidence of enteropathy-associated T-cell lymphoma (EATL), infectious lymphadenitis, or malignancy was found, supporting a conservative, non-surgical management approach.

The patient was commenced on a strict, lifelong gluten-free diet with dietetic support. Nutritional repletion included enteral high-calorie nutrition, parenteral multivitamins, intravenous albumin, iron, folate, calcium, and vitamin D. Systemic corticosteroids (oral prednisone 1 mg/kg/day) were initiated to control inflammation, given the presumed immune dysregulation and absence of infection. He was monitored closely for infectious complications, and imaging follow-up was scheduled.

At three months, clinical improvement was evident with a 5 kg weight gain, normalization of bowel habits, and improved serum albumin (31 g/l). Anti-TTG IgA titers declined, and CT imaging showed stable lymphadenopathy without progression or new cavitations. Bone marrow biopsy and PET-CT excluded occult lymphoproliferative disease.

At six months, the patient remained clinically stable with continued nutritional recovery and no signs of lymphomatous transformation. He remains under regular multidisciplinary follow-up.

## Discussion

CMLNS is a rare but severe complication of celiac disease, first reported by Hemet et al. in 1969 [1, 2]. It is associated with high morbidity and mortality, primarily due to severe malnutrition, infections related to hyposplenism, and, less commonly, intestinal hemorrhage [3]. Although its exact prevalence is unknown, fewer than 50 cases have been reported worldwide [4].

CMLNS usually occurs in adults with long-standing or refractory celiac disease and is characterized by persistent malabsorptive symptoms despite gluten withdrawal. Weight loss, diarrhea, and asthenia are common, and hyposplenism may coexist, increasing susceptibility to infections [3–5]. Lymphoma develops in about 20% of cases, mainly enteropathy-associated T-cell lymphoma, significantly worsening the prognosis [1]. Mortality rates remain high, reaching 50%, particularly in patients unresponsive to dietary treatment or with associated malignancy [2, 3].

Imaging is crucial for initial suspicion. The typical finding is mesenteric lymphadenopathy with central cavitation and fat-fluid levels, which, although suggestive, is not pathognomonic [5, 6]. Our patient had multiple pseudocystic mesenteric and ileocolic lymph nodes, raising concern for a neoplasm. Similar features may occur in tuberculosis, Whipple’s disease, or necrotic lymphoma [7], making histological confirmation essential.

Macroscopically, cavitated nodes often contain lipid-rich or necrotic material. Microscopically, the hallmark is a central cavity filled with acellular, eosinophilic debris surrounded by a rim of residual lymphoid tissue [3, 6]. In our case, histology revealed liquefactive necrosis with a peripheral mantle of lymphocytes and histiocytes, without atypia, granulomas, or organisms. Specific colorations excluded infection, while the absence of clonal markers ruled out lymphoma. These findings confirm the reactive and non-neoplastic nature of the process, emphasizing the diagnostic value of tissue examination.

Although serology and duodenal histology confirmed celiac disease in our patient, other conditions can mimic or coexist with CMLNS. Structural mimickers include tuberculous lymphadenitis, lymphoma, and Whipple’s disease, which may show similar imaging features but differ in distribution, enhancement patterns, or histological findings. Functional causes of persistent malabsorption despite a gluten-free diet such as microscopic colitis, pancreatic insufficiency, or small intestinal bacterial overgrowth should also be considered. Refractory celiac disease, particularly type II, poses a risk for ulcerative jejunitis and enteropathy-associated T-cell lymphoma. In our case, infectious etiologies were excluded, and histology confirmed typical cavitated lymph node architecture without malignancy, supporting the diagnosis of CMLNS [7, 8].

The pathogenesis remains debated; proposed mechanisms include chronic antigenic stimulation and immune-mediated vascular injury leading to ischemic necrosis [6, 9]. Management relies on strict lifelong gluten-free diet and aggressive nutritional support. Corticosteroids may be considered in refractory celiac disease or severe inflammatory presentations after failure of a strict gluten-free diet, initiated once lymphoma is excluded, and used for a limited duration due to uncertain benefit and potential risks [1, 6]. In our patient, dietary therapy, nutritional rehabilitation, and corticosteroids led to significant clinical improvement. At the 4-week follow-up, clinical stability allowed corticosteroids to be tapered and then fully discontinued. Six-month imaging remained stable, with no signs of lymphomatous transformation.

For comparison with previously described presentations, the table provides an overview of reported case reports and case series of CMLNS (Table 1).

**Table 1:** Published case reports and series of cavitating mesenteric lymph node syndrome associated with celiac disease.

First author	Year	Study type	No. of cases	Diagnostic approach	Peculiar pathological features
Hemet et al.	1969	Case report	1	Surgical biopsy	Central cavitation of mesenteric lymph node with necrotic debris; residual atrophic lymphoid rim
McBride et al.	2010	Case report + review	1	CT + surgical biopsy	Cavitated lymph nodes with fat–fluid levels; acellular eosinophilic material surrounded by residual lymphoid tissue
Rodríguez-Sánchez et al.	2011	Case report	1	CT, VCE, surgical excision	Central fibrino-necrotic cavity with lipid vacuoles and xanthomatous macrophages; thin peripheral rim of atrophic lymphoid tissue; no lymphoma
Echavarria et al.	2013	Case report (abstract)	1	CT + FNA	Central necrosis with surrounding fibrosis and lymphoplasmacytic aggregates
Ruch et al.	2019	Case series + review	4 (38 total reviewed)	CT, MRI, surgical biopsy	Pseudocystic lymph nodes with lipid-rich necrotic material; preserved nodal architecture; absence of granulomas or malignancy; occasional calcifications
Öner et al.	2024	Case report (radiological focus)	1	CT, MRI	pathology not detailed
Tinguria	2023	Review	—	Histopathology synthesis	Central cystic cavity with eosinophilic acellular material; residual reactive follicles and sinuses.
Present case	2026	Case report	1	CT + laparoscopic excision	Liquefactive necrosis forming cavitated lymph nodes; dense lymphohistiocytic rim

## Conclusion

CMLNS is a rare and severe complication of celiac disease that requires early recognition and multidisciplinary management. Prompt initiation of a gluten-free diet and nutritional support is essential to improve prognosis.
